# E2EE enhanced patient-centric blockchain-based system for EHR management

**DOI:** 10.1371/journal.pone.0301371

**Published:** 2024-04-01

**Authors:** Alaa Haddad, Mohamed Hadi Habaebi, Elfatih A. A. Elsheikh, Md. Rafiqul Islam, Suriza Ahmad Zabidi, Fakher Eldin M. Suliman

**Affiliations:** 1 Department of Electrical and Computer Engineering, International Islamic University Malaysia, Kuala Lumpur, Malaysia; 2 Department of Electrical Engineering, College of Engineering, King Khalid University, Abha, Saudi Arabia; Jazan University Faculty of Computer Science, SAUDI ARABIA

## Abstract

To secure sensitive medical records in the healthcare clouds, this paper proposes an End-to-End Encryption (E2EE) to enhance a patient-centric blockchain-based system for electronic health record (EHR) management. The suggested system with a focus on the patient enables individuals to oversee their medical records within various involved parties by authorizing or withdrawing permission for access to their records. Utilizing the inter-planetary file system (IPFS) for record storage is chosen due to its decentralized nature and its ability to guarantee the unchangeability of records. Then an E2EE enhancement maintains the medical data integrity using dual level-Hybrid encryption: symmetric Advanced Encryption Standard (AES) and asymmetric Elliptic Curve Cryptography (ECC) cryptographic techniques. The proposed system is implemented using the Ethereum blockchain system for EHR data sharing and integration utilizing a web-based interface for the patient and all users to initiate the EHR sharing transactions over the IPFS cloud. The proposed system performance is evaluated in a working system prototype. For different file sizes between 512 KB to 100 MB, the performance metrics used to evaluate the proposed system were the time consumed for generating key, encryption, and decryption. The results demonstrate the proposed system’s superiority over other cutting-edge systems and its practical ability to share secure health data in cloud environments.

## Introduction

Communication technologies provide a quick and effective way to transfer data and resources in the modern digital environment. Many industries, including healthcare, have experienced rapid growth as a result of these technologies.

Many healthcare institutions are finding themselves obligated to migrate their healthcare operations and data storage to cloud-based platforms owing to the advantages presented by cloud computing [1, 2]. The worldwide cloud computing healthcare industry has received a significant boost from the COVID-19 pandemic, with a projected growth of $25.54 billion anticipated from 2020 and 2024 [3]. Cloud computing offers the capability to effectively handle, share, secure, and save electronic health records (EHRs), medical images, pharmaceutical information systems, and laboratory information systems.

The Protocol which allows computers all over the world to store files and serve them as a portion of a peer-to-peer network is IPFS. IPFS defines as The Interplanetary File System, it is distributed file storage protocol that allows any computer in the network to install IPFS settings and start hosting the files and serving them. In other words, IPFS is a peer-to-peer network of nodes that collaborate in storing and sharing information, effectively managing a cloud storage service. The distinction between cloud storage and IPFS storage. We can store internet data in the cloud by using a provider who manages and operates data as a service. The data can then be retrieved on demand. while IPFS introduces storage across a network of storage providers rather than a centralized provider such as Google Cloud as we use it in the proposed study.

Furthermore, patients will receive better care thanks to current health records and continual communication between various healthcare professionals [4]. Sharing medical data could offer workable options to increase public information accessibility, ease public access to healthcare management, and provide continuing medical reporting. Telemedicine, for instance, enables the monitoring of vital statistics and delivery of healthcare services while leveraging information and communication technology in situations where the clinicians and the patients are not in the same location [5]. Although there are several issues with health care, such as privacy and accessibility, the development of technologies to treat medical issues has so far advanced swiftly. Electronic health data must be safely shared across networks to preserve data integrity and patient privacy. In order to guarantee data security and privacy services and avoid the disclosure of electronic health data in communications, an encryption system is frequently required as a particular device. In general, encryption methods encrypt data before it is stored in the cloud, preventing even a cloud service provider (CSP) from being able to access it.

The key is essential to cryptography and one of the trickiest things to manage. It is liable in law for any damage that might be sustained during the encryption and decryption procedures. The key that is used to encrypt and decrypt data must indeed be kept secure [6]. When the key is to be stored, a problem arises. Several common cryptographic techniques have been used in a variety of applications to store data in an encrypted form [7–10] according to a variety of works. The issue of protecting shared electronic health data remains despite all these rules, guidelines, and efforts [11]. This study suggests a healthcare record system that integrates cryptographic approaches in order to protect the privacy and confidentiality of sensitive and private health Information. Two encryption techniques are used by the system to ensure that healthcare data is kept more confidential. This work suggests a hybrid AES and ECC strategy for cloud storage data security that does not need a third party. The proposed hybrid approach (AES-ECC) is used to effectively maintain system security when using cloud storage [12]. The major goal of this hybrid strategy is to shorten the time required to maintain system security while reducing the key size of the data.

The main contributions of this research are:

- A blockchain-based patient-centric system is designed and implemented to control and authorize access to patients’ electronic health records by verifying information and maintaining data integrity.
- A hybrid E2EE encryption approach is used for data storage and transmission to and from the cloud that ensures data confidentiality and privacy.
- The proposed system was validated against other pertinent works for various timings of encryption, decryption, and key generation.

The subsequent sections of this paper are organized in the following manner. Related Work furnishes an overview of prior endeavors within the field. The specifics and process flow of the suggested system are presented in Methodology of the proposed Framework. In comparison to other relevant research, Result Analysis and Discussion shows the outcome and performance of the proposed investigation. The paper is concluded in Conclusion followed by suggestions to future work.

## Related work

(SeDaSC) has been presented for group data [13]. To safeguard against malicious attacks, uphold data confidentiality, facilitate secure data sharing without necessitating re-encryption, and control access, SeDaSC employs symmetrical encryption. The cryptographic server, acting as a reliable third party within SeDaSC, oversees encryption and decryption processes. Jana et al. describe a powerful multi-level data encryption system [14]. The suggested method uses the ECC technique to provide double authentication for cloud security [15]. It uses the quick symmetric AES method to encrypt the key and is a robust public-key cryptosystem with low computing complexity. A security architecture was presented by [16] for the safe transfer of medical data from healthcare facilities via the health cloud system. The proposed health cloud architecture employs an ECC-based cryptographic system for secure sharing. The TP-CS controls how encryption and decryption procedures are done. This model is evaluated by times of key generation, file encryption, file decryption, also on file uploading and downloading. In order to achieve guaranteed deletion in cloud storage, Bentajet et al. In [17] suggested a structure for how to implement a function prototype based on lightweight access control depends on cryptography based on IS (identity-based cryptography). The proposed approach also allows for key delegation and revocation. in the emergency session, Acute care teams have permission to access the patien’’s medical records (EMR) according to the dynamic revocable access control system known as the AC-AC protocol, which was developed by [18]. A hybrid encryption system called AC-AC merges ciphertext-policy attribute-based encryption and dynamic index-based symmetric searchable encryption [19]. Building upon the foundation of the MicroSCOPE protocol [20], the new protocol put forth facilitates the bestowal and removal of access rights for healthcare practitioners involved in an acute care unit, achieved through the utilization of scope values. The attending medical team is empowered to introduce a fresh team during emergency sessions through AC-AC algorithms, aligning with the temporal constraints of acute care scenarios. Employing ACAC, a team also holds the capability to rescind access permissions from another team that has fulfilled its responsibilities.

## Methodology of the proposed framework

With our proposed system, we built a permissioned architecture on top of an Ethereum Blockchain framework that gives patients full control over their information while also preserving their privacy, durability, and security.

Other blockchain platforms were evaluated, including Hyperledger and Corda, but Ethereum emerged as the most suitable for our application in many aspects:

- Smart Contract Functionality: Ethereum’s robust smart contract capabilities align seamlessly with our system’s requirement for secure and programmable transactions.
- Decentralization: Ethereum’s established decentralized network enhances the resilience and reliability of our healthcare blockchain system.
- Community and Ecosystem: Ethereum’s active developer community and extensive ecosystem provide ongoing support and a wealth of resources for our project.
- Interoperability: Ethereum’s compatibility with existing standards promotes smooth integration with healthcare infrastructure and systems.

Medical data is stored in the blockchain as hashes, while extensive medical data is placed off-chain in IPFS, a design that upholds the scalability and efficiency of the proposed system. The smart contract (SC) is defined as a protocol chain code, responsible for managing EHR access control based on patient-centric authorization. This SC is employed to establish a role-based access control chain code for recognized stakeholders. In contrast to incentive-based mining, this protocol prioritizes ensuring uniform access for all users. The role-based unique ID is initiated upon stakeholder registration. Through this methodology, each user possesses both public and private keys for the storage and transmission of medical data. In this process, the patient’s medical record is initially generated by the doctor, followed by the patient encrypting the record and storing it in IPFS, while simultaneously retaining the IPFS hash value on the Ethereum blockchain.

Doctor can view/ edit the patient’s medical record, based on patient-centric permission access, the patient can give or revoke the permission to access on own’s medical record.

The doctor has permission access to the patient’s medical record which is called IPFS temporary view or patient-centric access to EHR, then the patient commits the update on his medical records by doctor’s private and public keys and stores it in IPFS. in this way, we ensure the proposed system runs an interoperability mechanism. the patients have the right to manage their medical records and can share the permission only for the relevant stakeholders, while the doctor session will end before the hash value was committed, this revokes any access to EHR without granting permission from the patient and preserving data privacy in the proposed system.

Smart contracts are generated in the backend for various healthcare functions, and the role-based access control implemented in this framework serves to protect patient privacy. Additionally, in comparison to the existing system, our suggested solution offers enhanced scalability and interoperability features.

### Background of the proposed system

**Fig 1:**
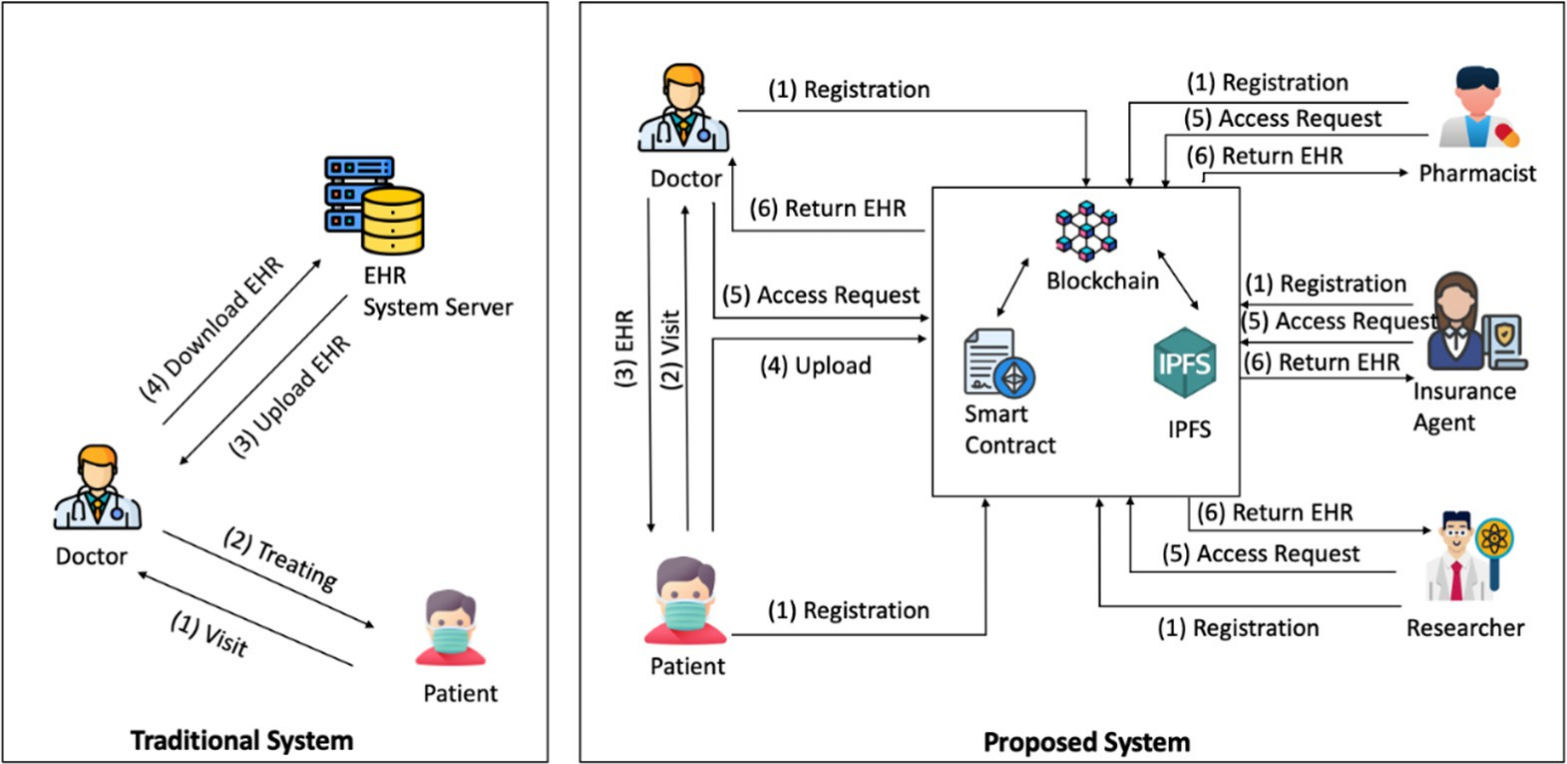
Overview of the traditional and the proposed solution.

The following Fig 1, shows the general layout of the proposed system and access control for exchanging EHRs utilizing blockchain technology. To maintain privacy, the EHR data will be encrypted using the patient secret key and saved in IPFS. As a result, a public key is used to encrypt the secret key. The essential data would then be saved on the blockchain for an authentication procedure, along with the encryption key. The EHR owner or other users, such as healthcare professionals, would have access to it.

We, therefore, suggest a blockchain-based security framework for sharing EHRs to protect patient privacy and guarantee data integrity in order to satisfy the requirements for blockchain in shared EHR systems. The suggested system and the conventional EHR system are contrasted in Fig 1.

In the conventional EHR system:

1. The patient sees the physician (Doctor).
2. The patient is then given medical attention by the doctor.
3. The doctor uploads the EHR to the server following treatment.
4. The doctor can download the EHR for later use.

Our strategy might be described as revolutionary secure electronic health records for patients that they can privately share. where the patient can independently manage, download, and trade his or her EHRs.

In our proposed study:

1. To produce and save keys, all users must be registered with the system.
2. The patient seeks medical attention from a doctor at a hospital or other healthcare facility.
3. The patient acquires his electronic health records, which will contain his private health information and be created following the assessment.
4. The patient submits the healthcare data to the blockchain and uploads the encrypted files straight to IPFS.
5. The user asks for access to the file (which might be a researcher, pharmacist, or doctor).
6. The user obtains the original file from the IPFS after receiving the encrypted data.

As a result, only the patient and others involved in the access control procedure can view the patient’s EHR documents. Moreover, a doctor is only permitted to look through the EHR of patients that they have already seen. Last but not least, a doctor or other users can only access an EHR if the owner has given them permission to do so.

The encrypted text in the electronic health record is being attempted to be decoded. Health records may be taken, changed, or falsified by a malevolent enemy. IPFS and the data requestors will agree to derive the EHR’s plain text. The security targets are as follows according to the threat model:

1. Data privacy: The original EHR of the owner cannot be revealed to unauthorized people.
2. Data authenticity: The patient’s EHR can be verified by those who have access to the data.
3. Integrity: Patient electronic health records (EHRs) can be saved securely to prevent tampering.
4. Data confidentiality: Patient electronic health records are kept secret from outsiders and stored securely.
5. Customizable access control: Patients have freedom to select their preferred EHRaccess methods, with only authorized individuals being granted permission.
6. Authentication: Users must first authenticate themselves in order to access EHR [15].

Algorithm 1 is used to enable patients to grant access to their health record to doctors through the use of Patient Centric EHR Smart Contract (PCEHRM-SC). This algorithm ensures that only specific fields of the health record are viewed and updated, rather than granting unrestricted access to the entire record. The patient-centric view is generated, and a session key (Sk) is created for use by both the patient and the doctor during the session. The session key is encrypted using the public keys of the patient and the doctor. Algorithm (1) calls the create_Update_HR() function to initiate the update of the health record. The doctor’s and patient’s session keys are decrypted, and the modifications are uploaded into the updated patient-centric view (UP_Pacenvn). Once the update is completed, the health record HR_n_ is saved in IPFS after the patient approves the changes. The Sk and Pacvn are then terminated, and the IPFS generates the health record hash value HRn_hash, which is saved in blocks within the Ethereum blockchain.

**Algorithm 1: System_Function ()**:

**     Input**: Doctor D_n_, with their public key Dpubk_n_, with their Private key Dprk_n_, with session key S_k_ of HR_n_ Health_Record. Patient Pa_n_ with their Public key Papubk_n_, and Private key Paprk_n_.

**Output**: **Boolean** (True or False)

1. Function for storing and updating health records

2. **For** user U have Access permission to HR

3. Check PCEHRM-SC

4. **If** (permission = = “Grant” && role = = “Doctor”) then

5.    Create Pacenv_n_ for HR_n_ in IPFS

6.    Pacenv_n_→ Decryption (Encryption (HR_n_))

7.    Create Sk

8.    send Encrypted (Papubk_n_ (Sk), Dpubk_n_ (S_k_), Pacenv_n_ (Sk)) to Pa_n_,

9.    D_n_ and Pacenv_n_.

10.   create_Update_HR ()

11.     HR_n _→ [(Decryption Papubrk_n_ (Encrypted Papubk_n_ (HR_n_)) + Encryption (UP_Pacenv_n_)]

12.  Pa_n_ → Commit (IPFS (HR_n_))

13.  IPFS → HR_n__hsh

14.  HRn_hsh → Ethereum Blocks

15.  **Return** True

16.**Else**

17.   Permission = Deny

18.   **Return** False

19.**End** **if**

20.**End** **For**

21. **End** Function

### Access control

Guarantees the privacy and accuracy of EHR. Only authorized healthcare professionals and patients should be able to access medical information on our suggested system. Patients should control how their data is collected and who has access to it. These access decisions are made by the patient listed in the smart contract. The smart contract will deny any access requests from unauthorized parties, and the system will be shut down as a result.

The patient’s consent and approval access is required before the user can retrieve health data. The only individual who can add another person to view the patient’s record is the patient. To ensure that the doctor has access to the patient’s data, the smart contract checks the access only before receiving the health data. If the doctor doesn’t have access control, the system delivers a bogus message and ends the session.

Scenario

**Fig 2:**
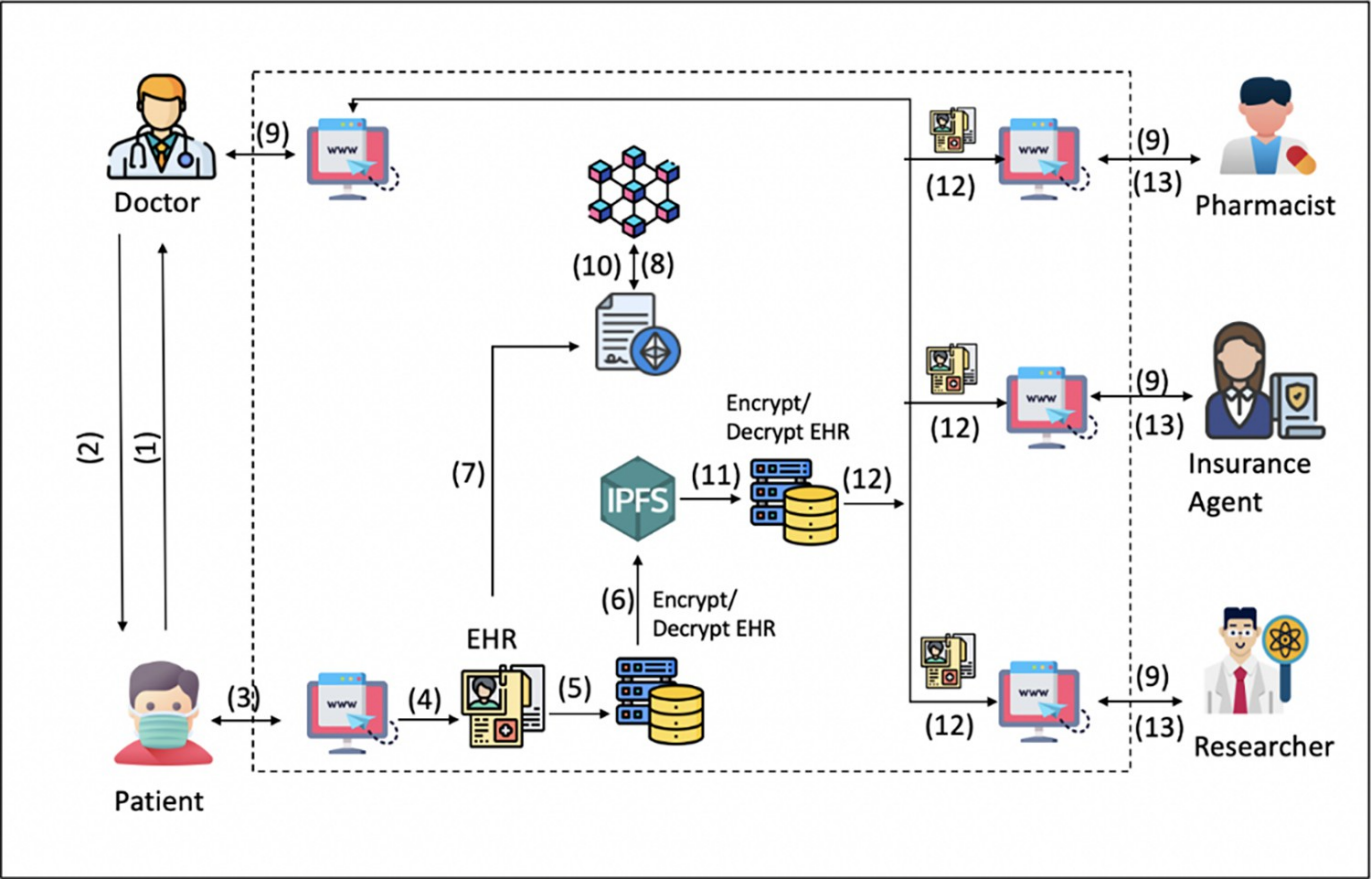
Interactions in the proposed system.

To communicate with the system, all actors use the web portal. To produce and store keys, each user needs to be registered with the system. In Fig 2, several interactions are shown.

1. Patients see doctors in hospitals or other healthcare facilities.
2. The patient receives access to his or her electronic health records, which include any private health information created following encounters with the doctor.
3. The patient enters his user ID and password to access the portal.
4. The patient adds the system with his EHR.
5. The patient adds a user’s access rights in accordance with the user’s role to his or her EHR.
6. The EHR will be encrypted and posted to the IPFS in encrypted form.
7. The patient uploads the key that has been encrypted along with other data to the blockchain.
8. All requests for transactions are logged to the blockchain [21].
9. In order to retrieve EHR, users submit an access request to the system with the necessary data.
10. The smart contract authenticates the user with access and decrypts the encrypted key.
11. Pulls encrypted EHR from IPFS and decrypts it.
12. The user, a doctor, downloads and consults the EHR.
13. Depending on his or her access privileges, the user can modify the patient’s electronic health record.

### Hybrid encryption

Data is encrypted and decrypted using the same key in symmetric encryption. Although very quick, algorithms using this method are not as secure as those using asymmetric encryption. Asymmetric encryption is thought to be safer because it does not require key sharing, but it is slower and takes longer to complete. We decided to combine symmetric and asymmetric encryption as a result. Because symmetric encryption is needed to convert plaintext to ciphertext, hybrid encryption is necessary. This utilizes the speed of symmetric encryption. The symmetric-key is encrypted using the asymmetric key to take advantage of the security of asymmetric encryption, making sure that only the intended recipient can decrypt the symmetric key.

### ECC (Elliptic Curve Cryptography)

ECC is a widely recognized cryptographic method designed to safeguard data from unauthorized access through the use of asymmetric key encryption. This technique relies on pairs of public and private keys to ensure ECC’s security. ECC employs two-dimensional fields encompassing both binary and prime fields. Implementing this cryptographic approach greatly enhances security against hacking attempts due to its intricate operations and the establishment of a relationship between binary and prime fields, thereby preventing unauthorized comprehension

ECC’s distinct characteristic lies in its compact key size. An ample number of points play a pivotal role in determining the suitable field for applying cryptographic measures to data for enhanced security. The initial field operation involves selecting the primary number and subsequently generating a larger number based on data ranging from 0 to Z. ECC finds specific use in key generation tasks, streamlining the complexity of operations involved. ECC’s enhancement is much greater than that of other cryptographic techniques due to its small key size.

### AES (Advanced Encryption Standard)

The symmetric key cryptography was developed by Joan Daemen and Vincent Rijmen, two Belgian cryptographers [22]. usually used to encrypt or decrypt large amounts of data more quickly. This is due to the fact that it uses the same key for both encrypting and decrypting operations rather than generating a new one.

The AES encrypts 128-bit blocks of data using 128-bit keys with 10 encryption rounds, 192-bit keys with 12 encryption rounds, or 256-bit keys with 14 encryption rounds [23]. It has been demonstrated to have a higher level of security than DES or 3-DES, come with a larger key size, and encrypt communications more quickly [24]. The following methodical processes are used in the encryption and decryption operations: To decode it, perform byte substitution, shift rows, mix columns, add a round key, and finally the opposite of these operations.

ECC and AES are used in combination to create an efficient cryptographic technique for secure cloud storage (IPFS). The hybrid (ECC-AES) method is faster and has a smaller key size than using a single AES due to ECC’s small key size feature. ECC employs encryption and decryption key standards to establish a secure key system and reduce key size, making it ideal for use with AES in protecting data from unauthorized access [20]. Once the key size is determined, ciphertext is generated using AES for data encryption and decryption with the key generated by ECC.

**Fig 3:**
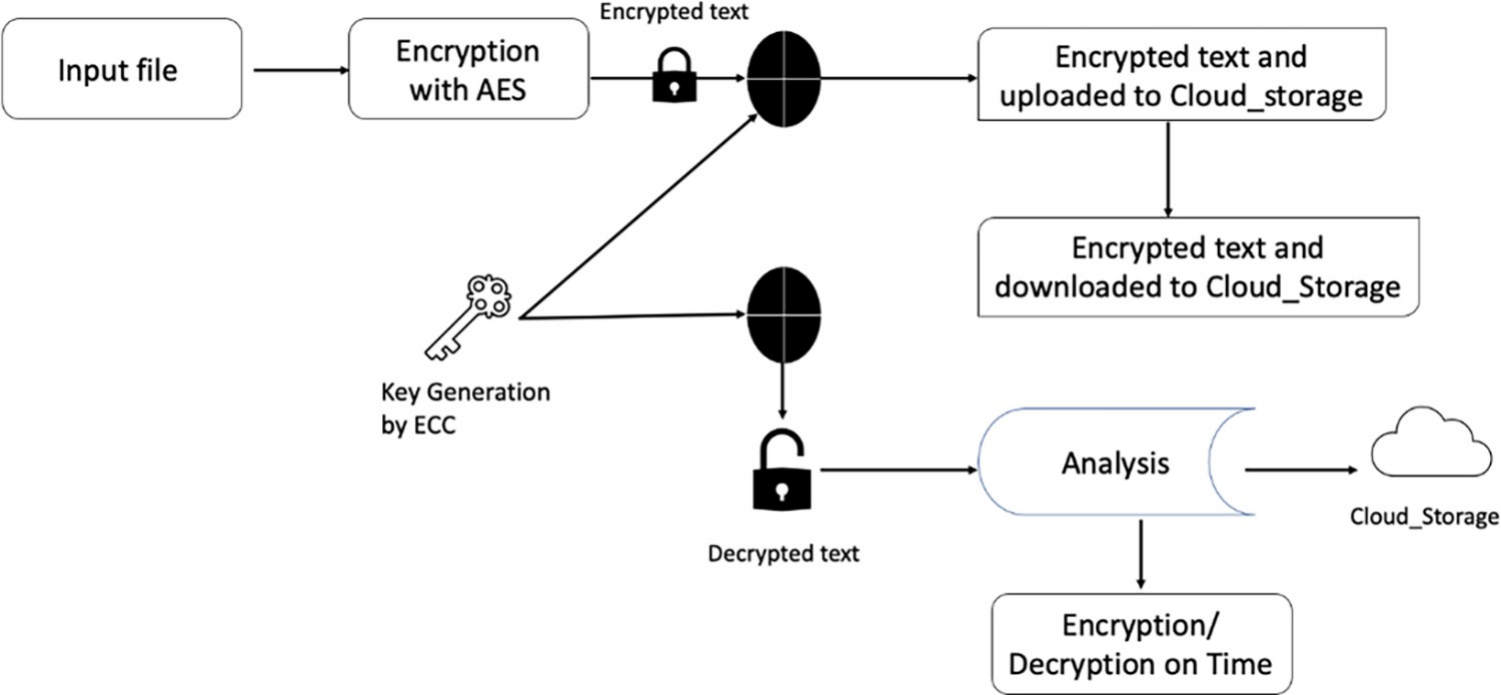
ECC and AES algorithm.

The proposed technique utilizes the combined effect of ECC and AES to create a secure system for cloud storage, which helps in reducing the size of secure data storage and memory space optimization and minimize computational complexity. In comparison to other cryptographic methods, the hybrid algorithm requires a medium amount of memory and takes less time to encrypt and decrypt data. Fig 3 shows the block diagram of the proposed algorithm.

The above diagram clearly shows how AES, in conjunction with ECC, effectively secures data stored in the cloud.

The proposed method is a novel approach for secure data transmission and storage. The system diagram provides an innovative solution for protecting user data during transmission to the server and secure storage of the encrypted data.

The novelty of the method can also be evaluated based on its computational cost and time. To prevent attacks, the user’s personal information is first encrypted using AES encryption when they upload the input file, making the text fully encrypted.

This ensures that the information is protected in case an attacker tries to access it. Additionally, even if an attacker does manage to obtain the encrypted file, they can’t decrypt it, thereby safeguarding the data from attacks

### Implement the hybrid encryption algorithm

**Fig 4:**
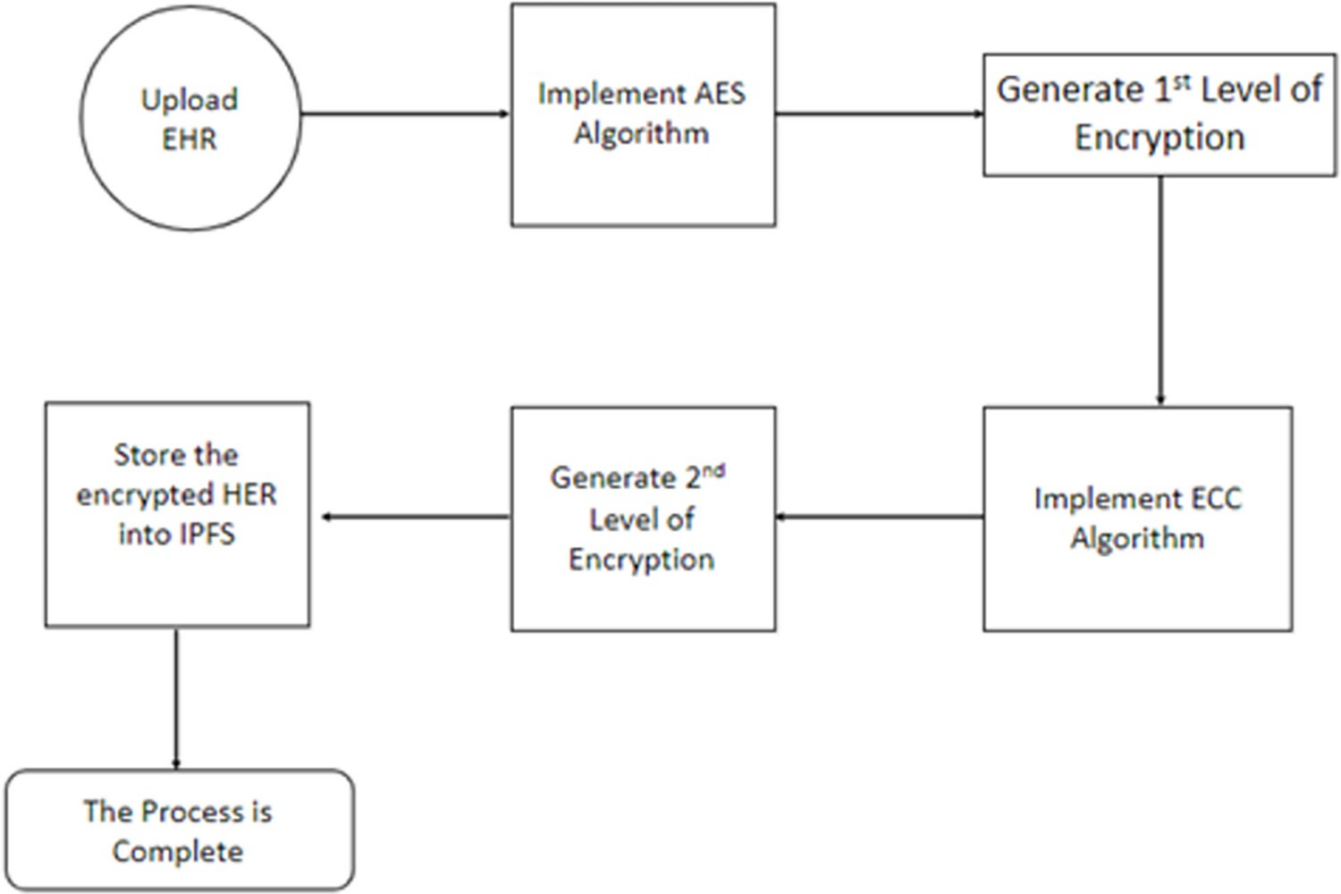
Hybrid encryption mechanism in the proposed system.

Our dual-level hybrid encryption mechanism, crucial for ensuring robust End-to-End Encryption (E2EE) within our blockchain-based system, is meticulously implemented as follows:

Users initiate the process by establishing unique keys and registering distinct accounts, as depicted in Fig 4. For each patient’s Electronic Health Record (EHR P), a 128-bit symmetric key (SK) is dynamically generated. This SK, crucial for data security, is produced through the SHA1PRNG pseudo-random number generation (_PRNG) method implemented by the SUN provider. The utilization of the hash function results in the creation of a reliable and unpredictable stream of random numbers.

Subsequently, the EHR is encrypted using the AES advanced encryption standard, bolstering data confidentiality. To enable secure data-sharing transactions, each blockchain user obtains key pairs—Public key (_PuK) and Private key (_PrK)—via hashing a random number (_RN) using the 256-bit SHA-1 hash method. The SK undergoes encryption, and the original EHR is signed using the key pair of elliptic curve cryptography, ensuring an additional layer of security through asymmetric key authentication.

Eqs (1) and (2) encapsulate the comprehensive process wherein EHR P generates all keys, employs the SK to encrypt the EHR, resulting in the creation of the ciphertext CEHR, and then utilizes the public key _PuK to encrypt the symmetric encryption key, forming the ciphertext key CK.


CEHR=EncEHR(IPFS,SK) (1)

CK=EncKey(SK,_PuK) (2)

The subsequent steps involve hashing the encrypted EHR (_MD) using Eq (3) and signing the hash using the private key, culminating in the creation of the digital signature (SIG). The patient’s EHR is then transmitted to the IPFS once the signature process is complete (Eq 4).


_MD=H(CEHR) (3)

SIG=(_MD,_PrK) (4)

Afterward, he or she transmits to the blockchain both SIG and encrypted keys (CK). Also, as mentioned before in acess control section, he or she transmits the smart contract access permissions. For instance, the system database contains the public keys for each user. The SK will be re-encrypted using the public key of B if A (the patient) wishes to share data with B (add B to the list of authorized users). B can use its private key to decrypt the data when it needs to access it. No one else is able to decrypt the data since only B has access to B’s private key. Algorithm 1, titled "Storing Data (Encryption)," succinctly outlines the encryption procedure for each EHR data, ensuring secure storage on IPFS and blockchain transactions. This process is integral to establishing predetermined access permissions within smart contracts, as delineated in the subsequent section.

Algorithm 1: Storing Data (Encryption)

1. Input: EHRi, Access control, _Puk, _Prk, Sk, SHA-2

2. Output: CEHR, CK, SIG

3. For each EHR data

4. CEHR → Encrypt_EHR(EHRi, SK);   i = [1;8]

5. CK→ Encrypt_key(SK, _Puk)

6. _MD→ H(Encrypt_EHR)

7. SIG→ H(_MD, _Prk)

8. Save _PuK in DB.

9. IPFS (CEHR).

10. BC (CK,SIG).

11. End For

12. End Function

In order to facilitate secure sharing of Electronic Health Records (EHR), the owner of the EHR establishes predetermined access permissions within smart contracts. These smart contracts encompass various aspects such as access permission, permissible actions, and granted rights (such as read and write capabilities).

The smart contract is activated as soon as the access need is satisfied, ensuring the accuracy and justice of the data sharing to carry out the associated procedure. The following two steps make up the EHR sharing process over the suggested system:

• Blockchain access:

- Access Request to EHR: Firstly, the user (U) starts to send a request (_Req) to exchange the transaction to the blockchain network. the following Eq (5) explains how each access request to EHR must include the ID which is the access target and EHR_i_ and _PrK. then the blockchain will receive the transaction request and authenticates the EHR user (U). and then the data transaction will be preserved in blockchains and the U is trusted

$$\_Req=(ID∥EHR_{i}∥\_PrK);\;i\in [1;8])$$ (5)

- Smart contract execution: Equation describes how the SK will be given to the user after being encrypted using the U’s private key if the Req is valid (6).

In the EHR sharing process, blockchain access involves an access request (_Req) initiated by the user (U) and subsequently validated by the blockchain network. Smart contract execution (Eq 6) ensures secure transmission of the SK to the user after encryption using the user’s private key.


Sk=DecCK(Ck,_PrK) (6)

-
**IPFS storage EHR sharing:**

The U will recover the EHR_i_ from the IPFS, as shown in Algorithm 2. The U then generates an _MD2 hash of the encrypted EHR to ensure its validity and integrity, as illustrated in Eq (7). The SIG is then decrypted using the EHR_P’s public key, and the outcome is displayed in Eq (8).


MD2=H(CEHR) (7)

DecSIG=(SIG,_PuK) (8)

The EHR user will decrypt the EHR and carry out its access action, as specified in Eq 7, if this decrypted _MD is match _MD2, this means the signature is valid (9). If not, the user can alert the system to the possibility that the data has been altered.


$$EHR_{i}=DecCEHR(CEHR,SK)$$ (9)

Algorithm 2, titled "Data Sharing (Decryption)," outlines the steps for EHR decryption, ensuring data integrity through the verification of the decrypted _MD matching _MD2. This process, facilitated by IPFS storage, guarantees the accuracy and security of the shared EHR.

Algorithm 2: Data Sharing (Decryption)

1. Input:  _Prk, Sk

2. Output: EHR

3. IF (_Req) is valid

4. Return  Sk → DecSK(CK, _Prk)

5.                Retrieve CEHR

6.                 _MD2→ H(CEHR)

7.               IF _MD = _MD2

8.               Return  EHR→ DecEHR(CEHR, SK)

9.               Else

10.               Return "Failure".

11. Else

12. Return "Failure".

13. End For

14. End Function

This comprehensive dual-level hybrid encryption mechanism not only safeguards patient data within the blockchain but also ensures secure and controlled sharing, underpinning the robust security architecture of our proposed healthcare data management system.

### Cost implications of system implementation

The implementation of our blockchain-based EHR management system involves considerations for both setup and operational costs. In terms of setup costs, the initial investment includes the establishment of the Ethereum blockchain infrastructure, incorporating nodes, implementing consensus mechanisms, and deploying smart contracts. Security measures, such as firewalls and encryption tools, are crucial for safeguarding sensitive patient data, contributing to the initial setup expenses. Additionally, development and integration efforts to seamlessly embed the system into existing healthcare infrastructure constitute a notable portion of the setup costs.

On the operational front, ongoing expenses include the maintenance of the blockchain infrastructure, ensuring its security, efficiency, and compliance with evolving standards. Personnel costs for skilled professionals to monitor the system, handle technical issues, and ensure smooth operation are ongoing considerations. The execution of smart contracts on the Ethereum network incurs transaction costs, and decentralized data storage on platforms like IPFS contributes to operational expenses.

## Result analysis and discussion

### Integration with existing healthcare infrastructure

Our proposed system seamlessly integrates with existing healthcare infrastructure and EHR systems:

1. Interoperability Framework: Adopts industry-standard interoperability frameworks, facilitating integration with diverse healthcare systems and ensuring seamless data exchange.
2. API Integration: Provides robust Application Programming Interfaces (APIs) for easy integration with existing EHR systems, allowing smooth data flow between our system and established healthcare platforms.
3. Data Standardization Measures: Incorporates data standardization protocols to ensure compatibility with prevailing healthcare data standards, promoting consistency and coherence in information exchange.

### Challenges and mitigation

1. Interoperability Challenges: Overcoming data standardization hurdles requires continuous collaboration with stakeholders, adherence to evolving standards, and implementing robust conversion mechanisms for different data formats.

In summary, our system prioritizes seamless integration with existing healthcare infrastructure by adhering to interoperability standards and implementing measures to navigate data standardization challenges.

### User interface design and usability

Our web-based system prioritizes intuitive design for diverse stakeholders. Usability testing involved patients and healthcare providers, ensuring:

- Intuitive Navigation: Clear and simple navigation empowers patients to manage EHRs effortlessly, while healthcare providers experience a streamlined workflow.
- Stakeholder-Focused Features: Tailored features cater to stakeholders’ specific needs, enhancing user experience for both patients and healthcare providers.
- Accessibility Considerations: Prioritizing accessibility, the interface accommodates diverse user needs with clear fonts, contrasting colors, and user-friendly layouts.
- Security Communication: Transparent communication of security measures instills user trust. The interface educates users on data confidentiality, reinforcing the system’s security features.

### Detailed assessment of the security features

#### Data integrity

Our system’s foundation lies in a dual-level hybrid encryption mechanism utilizing Advanced Encryption Standard (AES) and Elliptic Curve Cryptography (ECC). This not only secures patient data against unauthorized access but also plays a pivotal role in maintaining the integrity of EHR.

*1.1. AES Encryption*.

- Symmetric key generation: Each patient’s EHR (EHR P) undergoes encryption using a symmetric key (SK) generated by the SHA1PRNG pseudo-random number generation method. This 128-bit SK ensures a high level of randomness and strength.
- Unchangeability assurance: The unchangeability of records is preserved as the EHR is encrypted using the AES algorithm, ensuring the integrity of the data stored in the Inter-Planetary File System (IPFS).

*1.2. ECC Encryption*.

- Public and private key pairs: Each user in the blockchain system possesses key pairs (_PuK and _PrK) generated through the SHA-1 hash method. The public key is used to encrypt the symmetric encryption key (CK), providing an additional layer of security.
- Asymmetric key authentication: The original EHR is signed using ECC, enhancing the integrity of the data. The signature is created using the private key, ensuring that any alterations to the data are detectable.

### 2. Hash functions for integrity verification

To further fortify data integrity, our system employs hash functions to create message digests and verify the integrity of the encrypted EHR. The following steps outline the process:

*2.1. Hashing Encrypted EHR*.

Message Digest (MD): The encrypted EHR (CEHR) is hashed to generate a message digest (_MD) using a secure hash function (H). This process provides a unique identifier for the encrypted data, ensuring its integrity during storage and transmission.

*2.2. Signature Verification*.

Digital Signature (SIG): The MD is signed using the private key, generating a digital signature (SIG). This encrypted hash ensures the authenticity and integrity of the EHR during transactions on the blockchain.

our proposed system not only focuses on safeguarding EHR from unauthorized access but places a significant emphasis on ensuring the integrity of the data. The hybrid encryption mechanism, coupled with hash functions and digital signatures, creates a robust framework for preserving the accuracy and consistency of electronic health records.

### 3. Confidentiality

Utilizing dual-level hybrid encryption (AES and ECC), our system ensures robust confidentiality. Symmetric key encryption secures EHR data, while asymmetric key pairs and smart contracts enforce selective access control, limiting data exposure to authorized users. Secure key management and ongoing optimization efforts address potential vulnerabilities, solidifying our commitment to safeguarding patient information.

### 4. Authentication and authorization

Our system employs a dual-layer approach to Authentication and Authorization. Users generate key pairs through Elliptic Curve Cryptography (ECC), ensuring secure identity verification. Smart contracts establish predetermined access permissions, dictating permissible actions and user rights within the blockchain. Robust private key management and continuous monitoring uphold stringent authentication, while smart contract execution guarantees authorized data transactions, enhancing overall system security.

### 5. Data Recovery and system resilience

- Regular Backups: Implementing regular and automated backups of critical data ensures rapid recovery in case of data loss or corruption.
- Redundancy and Replication: Employing redundant data storage and replication mechanisms minimizes the impact of hardware failures, enabling swift restoration.

5.1. System Resilience against Cyber-Attacks.

- Real-Time Monitoring: allows for early detection of anomalies or potential cyber threats.
- Distributed Architecture: enhances resilience, making it challenging for attackers to compromise the entire system in one go.

### Metric times

For confirmation, we also compared the encryption and decryption times for our proposed hybrid method using various key sizes and with the already-in-use techniques (AES, DES). Several keys, including 64 bits, 128 bits, 192 bits, and 256 bits, were used for the tests. Using various keys, the proposed and current encryption techniques were tested for text data. Firstly, the following Table 1 shows the key generation time for asymmetric and symmetric in different sizes.

**Table 1:** Shows key generation time for ECC-AES in different size.

Key Size	Time in nanoseconds (Encryption)	Time in nanoseconds (Decryption)
64	2178500	3876199
128	2386100	4083799
192	2384900	4082599
256	2301300	3998999

**Fig 5:**
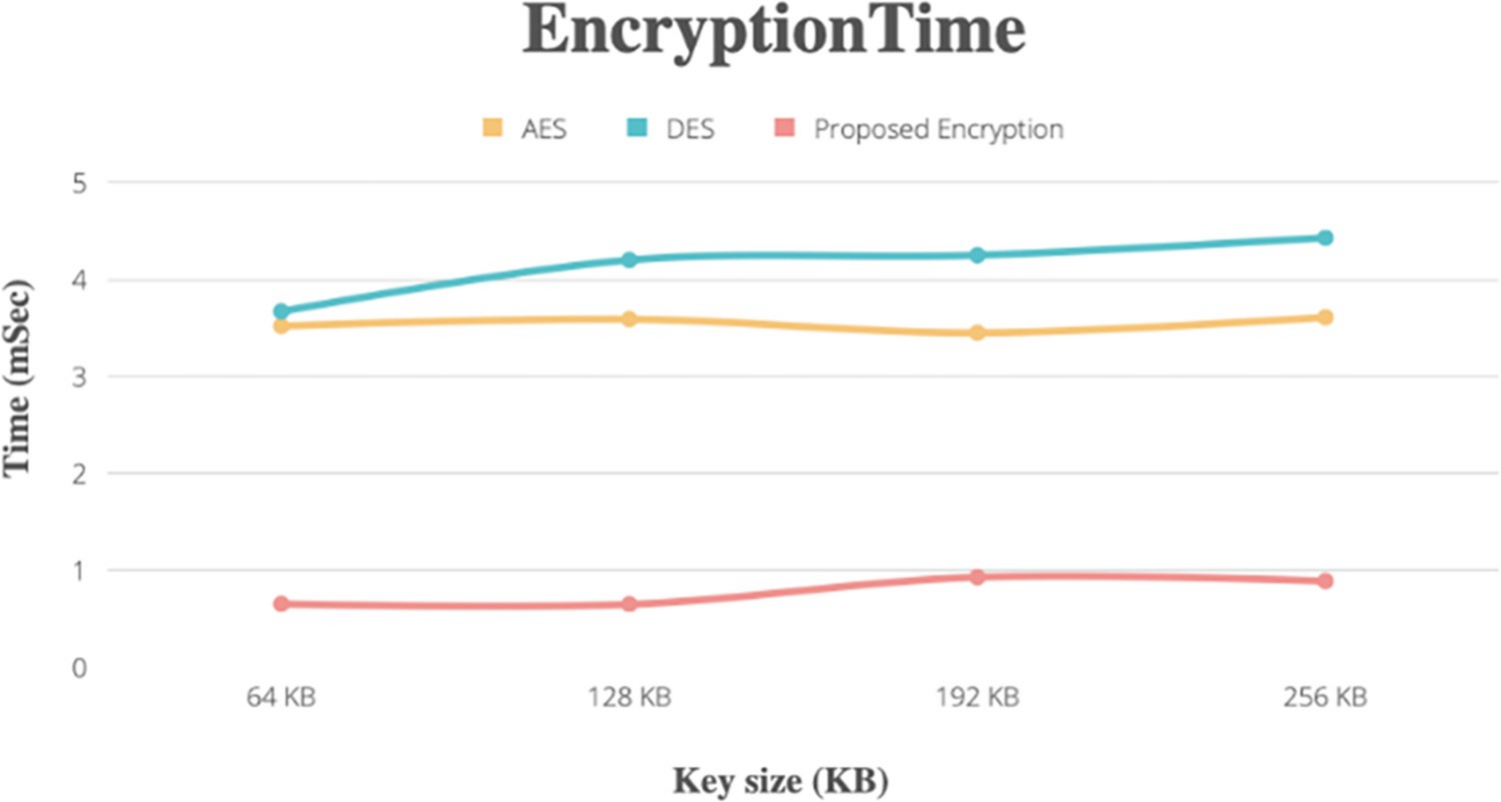
Compare time with different key sizes for the encryption process with other methods.

Fig 5 compares the times required for the encryption process of identical text data by the AES, DES, and hybrid methods. The hybrid ECC-AES model was found to take less time on key sizes in bits (key = 64, key = 128, key = 192, key = 256), but the other algorithms in use took more time on key sizes.

**Fig 6:**
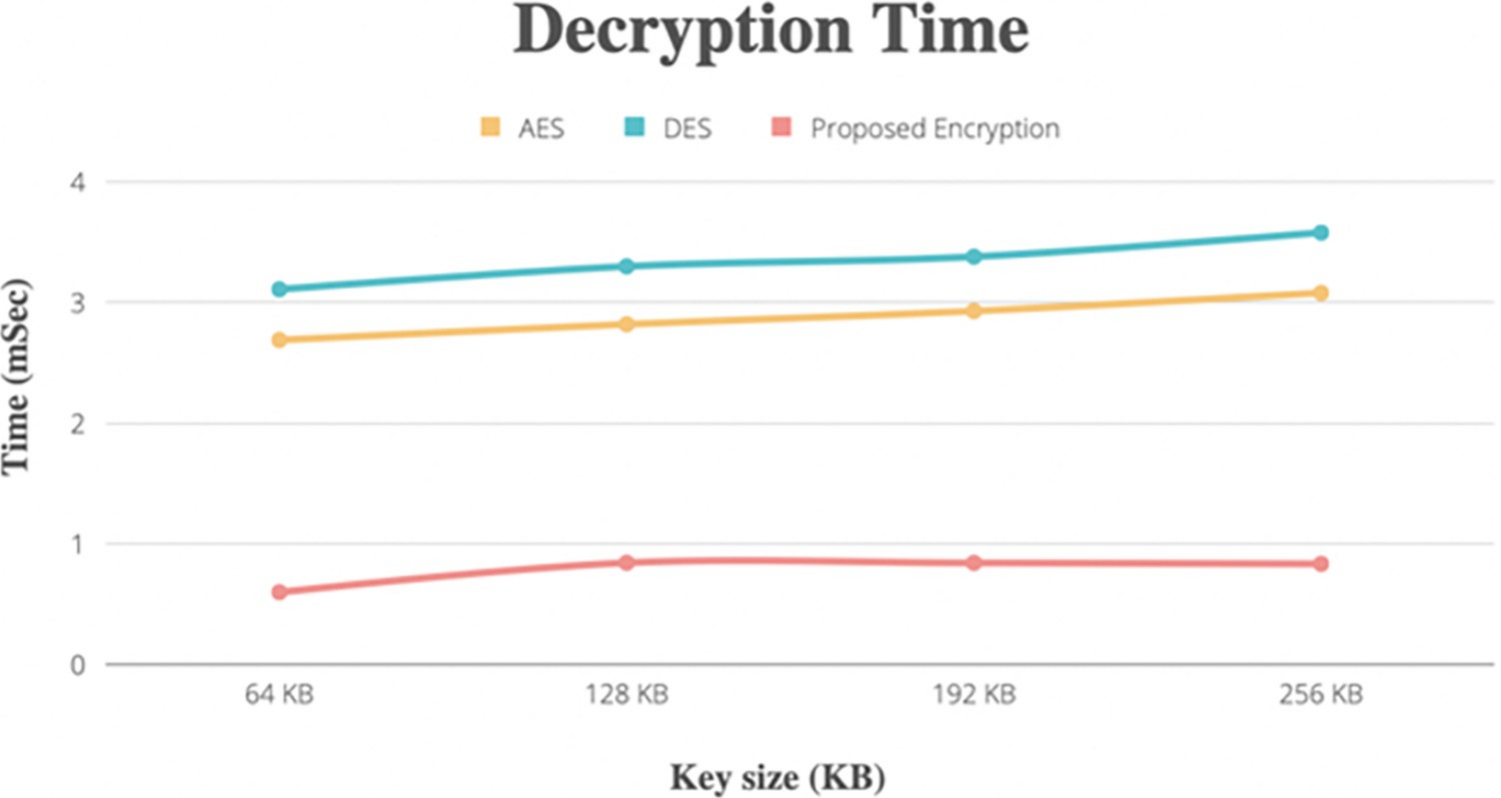
Compare time with different key sizes for the decryption process with other methods.

Fig 6 illustrates how long the decryption of the same text data using the AES, DES, and hybrid methods takes in comparison. The hybrid model was found to take less time on keys from key = 64 to key = 256 bits, but the two existing algorithms (AES, DES) spent more time on keys of those same sizes.

Figs 5 and 6 show how, in comparison to previous cryptographic algorithms, our scheme will aid in memory space optimization and minimize computational complexity. In comparison to other cryptographic methods, the hybrid algorithm requires a medium amount of memory and takes less time to encrypt and decrypt data.

Encryption time is the amount of time required to encrypt the health record based on the EHR owner’s request, whereas the decryption time is the amount of time required to decrypt the EHR based on the user’s request. Our proposed encryption and decryption methods save time around 74% in encryption and decryption process in execution time.

Fig 5 depiction of encryption and decryption times shows a consistent rise in time consumption with record size. This indicates that a file’s size affects how long it takes to encrypt and decode it.

**Fig 7:**
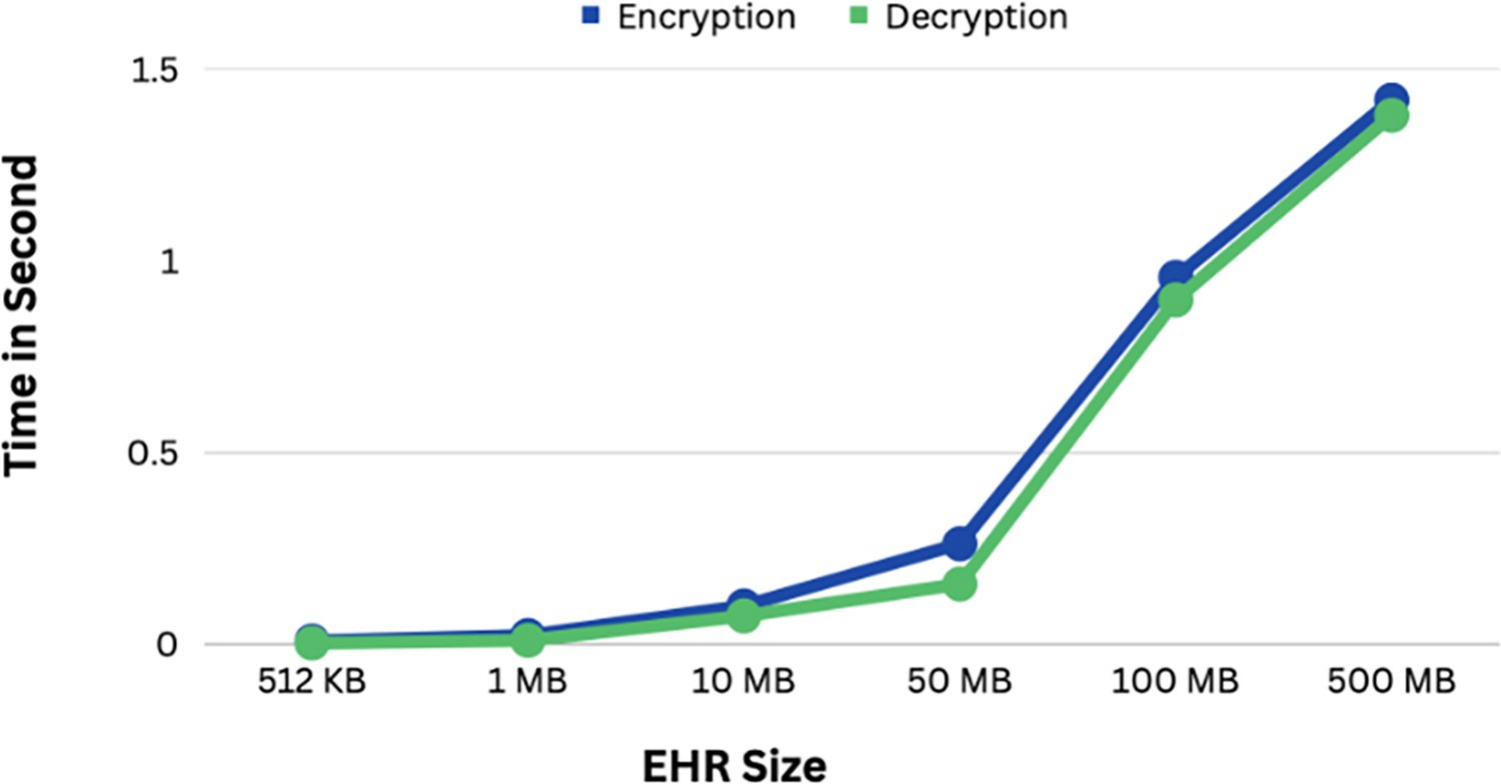
Encryption and decryption time consumption with different EHR sizes.

Fig 7 compares the encryption and decryption times required for blockchain operations with various Electronic Health Record (EHR) sizes, using Thin and Vaspongayya’s (2019) [25] approach and Boumezbur et al.’s (2022) [7] method. It illustrates how our study’s encryption and decryption times relate to the size of the EHR, the encryption and decryption times somewhat rise along with the EHR size. Moreover, the encryption and decryption processes take less than 1 second to complete when the EHR is around 100 MB in size. Even if the EHR is more than 100 MB, the extra time required is only around 1 second.

Table 2 compares the test results with the amount of time spent encrypting and decrypting data for different EHR file sizes.

**Table 2:** Compare encryption and decryption time with different EHR sizes.

File Size	Thin and Vaspongayya (2019)		Boumezbur I, et al. (2022).		Proposed system	
	En	De	En	De	En	De
512 KB	0.094	0.0064	0.0158	0.0027	0.0053	0.0017
1MB	0.101	0.0166	0.0452	0.0157	0.0126	0.0089
10 MB	0.152	0.069	0.075	0.058	0.0489	0.0227
50 MB	0.503	0.438	0.406	0.401	0.261	0.165
100MB	1.428	1.414	1.149	1.228	0.957	0.898

Our encryption and decryption efficiency are much better than those of Thin and Vaspongayya and Boumezbur I,et al. when the EHR is large. X-ray images are just examples of the numerous huge image files included in EHRs. Based on these comparisons, our technique is superior to earlier work on health record encryption and decryption.

**Fig 8:**
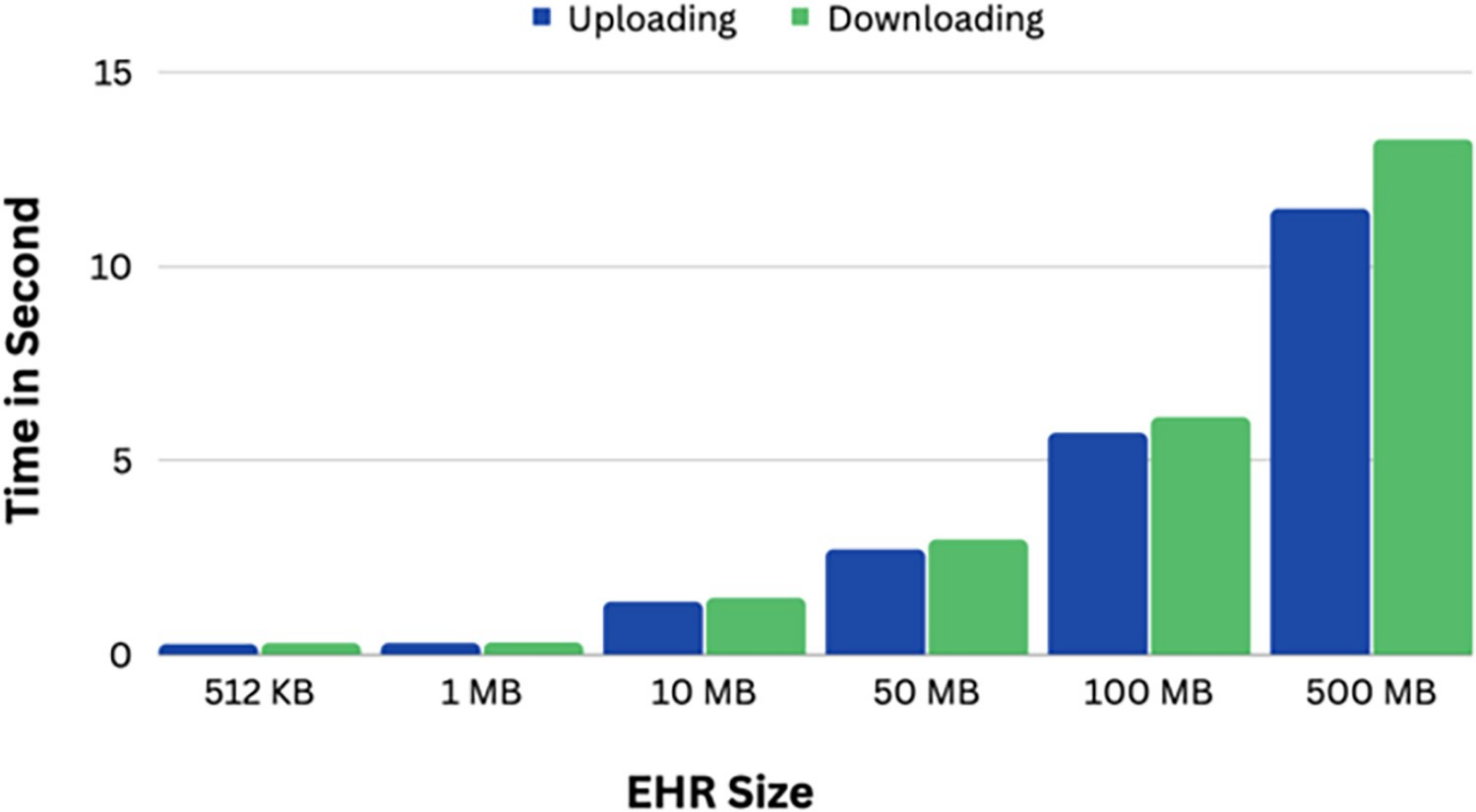
Time to upload and download different EHR size.

The length of time it takes to upload and download a health record to and from the IPFS is the basis for the system evaluation. The performance of uploading health records to the IPFS for various record sizes is shown in Fig 8. The graph below displays all of the constituent times. The size of a health record has a direct relationship with how long it takes to upload it to the IPFS. By stating tha’ the key’s calculation time is unaffected by’the file’s size, we can see that it has remained stable. However sometimes the record upload time only slightly increased, which can be related to th’ network’s status at the time.

In other hand, process of original health record that is downloaded from IPFS is the exact opposite of what is uploaded. Also, Fig 8 shows the outcomes of the IPFS download procedure and the decryption process. The download time continuously increases from 512KB to 500 MB as the record size does. At the smallest record size of 512KB, it takes only 0.285 seconds, but the larger record computation of size 500 MB significantly lengthens the download time to 13.26 s. The outcomes trend is therefore the same as the uploading process. Moreover, both download and decryption times are evolving.

Finally, To demonstrate the usefulness of our study, we compare the key generation time, record encryption time, record decryption time, record upload time, and record download time for different file sizes ranging from 512 KB to 500 MB. A comparison of our proposed system to other nearby works is also included, based on a set of security criteria such as integrity, privacy, access control, encryption, and key encryption.

The suggested system is shown in Table 3 along with the upload and download process response times for various studies, where "UP" and "DN" stand for "upload" and "download," respectively. The proposed system performs better than the other current approaches as a result. It provides superior upload and download performance, albeit the numbers may differ significantly depending on the speed of the internet connection and the size of the records. Table 3’s comparisons of response times for encryption and decryption show that the proposed system performs better than other approaches due to the lack of complex calculations. The suggested procedure is at least twice as quick as the other.

**Table 3:** Comparison of uploading and downloading time for encrypt/decrypt files.

File size	Hema and Kesavan (2019)		Boumezbur I,et al. (2022)		Proposed system	
	UP	DN	UP	DN	UP	DN
**512 KB**	0.80	0.82	0.32	0.35	0.26	0.285
**1 MB**	1.20	1.24	0.35	0.39	0.287	0.30
**10 MB**	5.60	5.68	1.82	1.89	1.35	1.45
**50 MB**	8.25	8.78	3.2	3.25	2.7	2.95
**100 MB**	16.35	18.98	7.69	8.01	5.70	6.1
**500 MB**	32.10	38.22	14.36	18.03	11.47	13.26

### Scalability assessment

- Performance Under Different Loads:

The proposed system exhibits commendable scalability, demonstrated through rigorous testing across various loads. From file sizes ranging between 512 KB to 100 MB, the system consistently maintains efficient key generation time as shown in Table 1, encryption, and decryption times as shown in Figs 5 and 6, respectively. Performance metrics underscore its ability to handle increasing data loads while ensuring timely processing.

- Limitations in Handling Large Numbers of EHRs or Concurrent Users:

Despite its robust design, the system recognizes potential hurdles when dealing with vast datasets. Processing times might exhibit incremental delays with notably larger files, as illustrated in Table 4. Moreover, the system faces constraints in terms of blockchain transaction throughput and smart contract overhead, especially under intense concurrent user interactions.

**Table 4:** System performance metrics.

No. of Transactions	Throughput (tx/s)
0	100
100	95
200	90
300	85
400	80
500	75

- Throughput Dynamics: The system’s throughput, depicted in Fig 7, showcases a gradual decrease as the number of transactions escalates. This trend signals potential challenges in efficiently handling an extensive volume of concurrent transactions.
- Smart Contract Overhead Analysis: Concurrent user interactions may lead to increased smart contract overhead, impacting the system’s responsiveness.
- The rise in smart contract execution time is a critical consideration for sustained system efficiency.
- Optimization Strategies:Given the identified limitations, future optimization efforts should explore parallel processing strategies.

Parallelization can enhance system scalability, addressing challenges associated with larger datasets and concurrent user demands.

## Comparison with traditional EHR systems

In comparison to traditional EHR systems, the proposed blockchain-based solution offers distinct advantages and challenges. The decentralized efficiency of blockchain reduces reliance on centralized authorities, potentially leading to long-term operational cost savings. Enhanced security features, including cryptographic techniques, may contribute to cost reductions associated with data breaches and regulatory fines.

However, challenges exist, particularly in the initial stages. The setup investment for blockchain integration can be higher than traditional systems. Staff training for blockchain technology and the transition from conventional EHR systems might entail additional costs.

## Legal and regulatory compliance overview

1. HIPAA Compliance: The system aligns with HIPAA regulations through robust security measures, including End-to-End Encryption (E2EE), safeguarding patient health data and ensuring confidentiality.
2. GDPR Compliance: Compliant with GDPR, the system prioritizes patient data ownership, transparent processing, and secure transactions, adhering to privacy-by-design principles.
3. Key Features: Emphasizes patient-centric control, secure data transactions, and continuous compliance monitoring through regular audits, adapting swiftly to evolving legal requirements.

## Future work

While our current long-term maintenance and support plan lays a strong foundation for system sustainability, there are avenues for future enhancements. Research and development efforts can focus on predictive maintenance models, leveraging artificial intelligence and machine learning to anticipate potential issues and optimize system performance. Additionally, exploring blockchain advancements and emerging technologies will contribute to the system’s continuous adaptation to the evolving technological landscape. Collaborations with industry experts and stakeholders can further refine the support plan, ensuring alignment with dynamic healthcare industry needs. Continuous monitoring of industry trends and regulatory changes will be integral for proactively integrating new features and maintaining the system’s efficacy in the ever-evolving healthcare landscape.

## Conclusion

The proposed system is based on patient-centric control, the patient has all rights to grant or revoke permission to access his/her record to save the data privacy. Furthermore, the proposed study enhances data security against third parties and prevents them from accessing the medical data without patients’ permission enabling patient-centric confidentiality, and integrity of the data. The proposed system features an E2EE cryptographic approach by implementing hybrid encryption combining symmetric and asymmetric encryption ECC-AES algorithms to ensure data security and integrity. we evaluated the proposed system based on the time consumed for generating key, encryption, and decryption for different file sizes between 512 KB to 100 MB. The results demonstrate the proposal’s superior performance compared to other cutting-edge systems and its practicality.

## References

[1] Low C, Hsueh Chen Y. Criteria for the evaluation of a cloud-based hospital information system outsourcing provider. Journal of medical systems. 2012 Dec;36:3543–53. doi: 10.1007/s10916-012-9829-z 22366976

[2] Poulymenopoulou M, Malamateniou F, Vassilacopoulos G. Emergency healthcare process automation using mobile computing and cloud services. Journal of medical systems. 2012 Oct;36:3233–41. doi: 10.1007/s10916-011-9814-y 22205383

[3] Shakeel T, Habib S, Boulila W, Koubaa A, Javed AR, Rizwan M, Gadekallu TR, Sufiyan M. A survey on COVID-19 impact in the healthcare domain: worldwide market implementation, applications, security and privacy issues, challenges and future prospects. Complex & intelligent systems. 2023 Feb;9(1):1027–58. 35668731 10.1007/s40747-022-00767-w PMC9151356

[4] Al-Issa Y, Ottom MA, Tamrawi A. eHealth cloud security challenges: a survey. Journal of healthcare engineering. 2019 Sep 3;2019. doi: 10.1155/2019/7516035 31565209 PMC6745146

[5] Xiao J., Liu X., Zeng J., Cao Y., & Feng Z. (2022). Recommendation of Healthcare Services Based on an Embedded User Profile Model. International Journal on Semantic Web and Information Systems (IJSWIS), 18(1), 1–21.

[6] Nguyen G. N., Le Viet N. H., Elhoseny M., Shankar K., Gupta B. B., & Abd El-Latif A. A. (2021). Secure blockchain enabled Cyber-physical systems in healthcare using deep belief network with ResNet model. Journal of parallel and distributed computing, 153, 150–160.

[7] Boumezbur I, Zarour K. Privacy-Preserving and Access Control for Sharing Electronic Health Record using Blockchain Technology. Acta Informatica Pragensia. 2022 Feb 17;11(1):105–22.

[8] Pugazhenthi A, Chitra D. Data access control and secured data sharing approach for health care data in cloud environment. Journal of medical systems. 2019 Aug;43(8):258. doi: 10.1007/s10916-019-1381-7 31264005

[9] Rajakumar MP, Ramya J, Sonia R. A novel scheme for encryption and decryption of 3D point and mesh cloud data in cloud computing. Journal of Control Engineering and Applied Informatics. 2021 Mar 26;23(1):93–102.

[10] Singh N, Singh AK. Data privacy protection mechanisms in cloud. Data Science and Engineering. 2018 Mar;3(1):24–39.

[11] Suresh D, Florence ML. Securing personal health record system in cloud using user usage based encryption. Journal of medical systems. 2019 Jun;43(6):171. doi: 10.1007/s10916-019-1301-x 31065802

[12] Lu J., Shen J., Vijayakumar P., & Gupta B. B. (2021). Blockchain-based secure data storage protocol for sensors in the industrial internet of things. IEEE Transactions on Industrial Informatics, 18(8), 5422–5431.

[13] Ali M, Dhamotharan R, Khan E, Khan SU, Vasilakos AV, Li K, Zomaya AY. SeDaSC: secure data sharing in clouds. IEEE Systems Journal. 2015 Jan 13;11(2):395–404.

[14] Jana B, Poray J, Mandal T, Kule M. A multilevel encryption technique in cloud security. In2017 7th International Conference on Communication Systems and Network Technologies (CSNT) 2017 Nov 11 (pp. 220–224). IEEE.

[15] Vinoth R., Deborah L. J., Vijayakumar P., & Gupta B. B. (2022). An anonymous pre-authentication and post-authentication scheme assisted by cloud for medical IoT environments. IEEE Transactions on Network Science and Engineering, 9(5), 3633–3642.

[16] Sri Vigna Hema V, Kesavan R. ECC based secure sharing of healthcare data in the health cloud environment. Wireless Personal Communications. 2019 Sep;108:1021–35.

[17] Bentajer A, Hedabou M, Abouelmehdi K, Igarramen Z, El Fezazi S. An IBE-based design for assured deletion in cloud storage. Cryptologia. 2019 May 4;43(3):254–65.

[18] de Oliveira MT, Dang HV, Reis LH, Marquering HA, Olabarriaga SD. AC-AC: dynamic revocable access control for acute care teams to access medical records. Smart Health. 2021 Apr 1;20:100190.

[19] Chen L, Lee WK, Chang CC, Choo KK, Zhang N. Blockchain based searchable encryption for electronic health record sharing. Future generation computer systems. 2019 Jun 1;95:420–9.

[20] Michalas A, Bakas A, Dang HV, Zalitko A. MicroSCOPE: enabling access control in searchable encryption with the use of attribute-based encryption and SGX. InSecure IT Systems: 24th Nordic Conference, NordSec 2019, Aalborg, Denmark, November 18-20, 2019, Proceedings 24 2019 (pp. 254–270). Springer International Publishing.

[21] Egala B. S., Pradhan A. K., Badarla V., & Mohanty S. P. (2021). Fortified-chain: a blockchain-based framework for security and privacy-assured internet of medical things with effective access control. IEEE Internet of Things Journal, 8(14), 11717–11731.

[22] Mendonca SN. Data security in cloud using AES. Int. J. Eng. Res. Technol. 2018 Jan;7.

[23] Feldman J, Misenar S, Conrad E. Eleventh Hour CISSP¬Æ: Study Guide. Syngress; 2016 Sep 3.

[24] Azad TB. Understanding XenApp Security. Securing Citrix Presentation Server in the Enterprise. 2008 Jan:259–316.

[25] Thin TT, Vaspongayya S. Blockchain-based access control model to preserve privacy for personal health record systems. Security and Communication Networks. 2019 Jun 25;2019.

